# Ultrastructural changes during the symbiotic seed germination of *Gastrodia elata* with fungi, with emphasis on the fungal colonization region

**DOI:** 10.1186/s40529-019-0280-z

**Published:** 2020-02-12

**Authors:** Yuan-Yuan Li, Shun-Xing Guo, Yung-I Lee

**Affiliations:** 1 College of Plant Protection/Beijing Key Laboratory of Seed Disease Testing and Control, China Agricultural University, Beijing, 100193 People’s Republic of China; 2 Institute of Medicinal Plant Development, Chinese Academy of Medical Sciences & Peking Union Medical College, Beijing, 100193 People’s Republic of China; 3 Biology Department, National Museum of Natural Science, 40453 Taichung, Taiwan; 4 Department of Life Sciences, National Chung Hsing University, 40227 Taichung, Taiwan

**Keywords:** Mycorrhiza, Mycoheterotrophic orchids, Phytophagy, Symbiotic germination

## Abstract

**Background:**

*Gastrodia elata* is a fully mycoheterotrophic orchid and has long been used in traditional Chinese medicine. The life cycle of *G. elata* requires an association with two different fungi-*Mycena* for seed germination and *Armillaria* for tuber growth. The association with *Armillaria* is representative of the phytophagous type of orchid mycorrhiza: the intracellular hyphae are lysed without forming condensed pelotons. However, whether the association with *Mycena* during seed germination belongs to the same type of orchid mycorrhiza is unknown.

**Results:**

Histological and ultrastructural studies revealed several notable features in different developmental stages. First, a thickened cell wall with papillae-like structures appeared during fungal penetration in the suspensor end cell, epidermal cells and cortical cells of germinating embryos. In addition, the formation of two distinctive cell types in the colonized region of a protocorm (i.e., the passage canal cell filled with actively growing fungal hyphae) can be observed in the epidermal cell, and the distinctive digestion cell with a dense cytoplasm appears in the cortex. Finally, within the digestion cell, numerous electron-dense tubules form a radial system and attach to degrading fungal hyphae. The fungal hyphae appear to be digested through endocytosis.

**Conclusions:**

The present study provides important structural evidence for the phytophagous type of orchid mycorrhiza in the symbiotic germination of *G. elata* with *Mycena*. This case demonstrates a particular nutrient transfer network between *G. elata* and its litter-decaying fungal partner.

## Background

Orchid seeds are minute, and most contain an undifferentiated embryo that lacks a well-defined endosperm (Arditti 1992). The few-celled embryo has small amounts of proteins and lipids and very little sugar (Harrison 1977; Arditti and Ghani 2000). Because of the lack of nutritional reserves, seed germination in nature completely depends on mycorrhizal fungi, which are believed to provide nutrients required for seed germination and protocorm development (Arditti 1967; Dearnaley 2007). There has been extensive research on symbiotic germination (Rasmussen 1995; Smith and Read 2008).

Previous studies of symbiotic germination have provided considerable information about the interaction between mycorrhizal fungi and orchid seeds, especially structural and ultrastructural changes (Burgeff 1909; Clements 1988; Peterson and Currah 1990; Uetake et al. 1992, 1997; Peterson et al. 1998; Chen et al. 2014). As the mycorrhizal fungi penetrate the embryo, they form hyphal coils, known as pelotons, enveloped by the plasma membrane in the host cell. Then, the pelotons collapse and undergo lyses, and the digested products are absorbed by the host cell. These studies focused mainly on green orchids, with a few studies on achlorophyllous orchids.

*Gastrodia elata*, an orchid used in traditional Chinese medicine, is widely distributed in many Asian countries, including China, Korea and Japan. The dry tuber of *G. elata* (known as *Tianma* in Chinese) has been used to treat many human illnesses, such as headache, vertigo, hemiplegia and infantile convulsions (Xu and Guo 2000) and has strong potential for treating Alzheimer’s and Parkinson’s disease (Manavalan et al. 2012). *G. elata* is a fully mycoheterotrophic orchid species. It sets up a symbiotic relationship with two different compatible mycorrhizal fungi during its life history. For seed germination, *Mycena* is recruited for stimulating germination and the early stages of protocorm development, then further development of tubers requires *Armillaria* (Xu and Guo 2000).

Previous studies identified two different histological types of orchid mycorrhiza: tolypophagy, occurring in most orchid species, and phytophagy, found in only a few mycoheterotrophic orchid species (Burgeff 1936; Rasmussen 2002). In *G. elata*, the association with *Armillaria* is representative of ptyophagic infection. In the inner cortex cells of *G. elata* tubers, intracellular hyphae of *Armillaria* are lysed without forming distinct hyphal coils (Wang et al. 1997). However, for symbiotic seed germination, the fungal partner *Mycena* forms distinct intracellular hyphal coils in the inner cortex cells of the protocorm, which suggests a tolypophagic relation (Fan et al. 1999).

Despite a reported association between *G. elata* and *Mycena* during symbiotic seed germination (Guo and Xu 1990; Xu and Fan 2001; Fan et al. 1999, 2001, 2002), information on serial structural changes in symbiotic seed germination with a defined time scale is still lacking. In this study, based on a defined time frame, we describe the histological and ultrastructural changes during symbiotic seed germination of *G. elata*. Better understanding the structural development in symbiotic seed germination can provide insights into the interaction between a mycoheterotrophic orchid and mycorrhizal fungi.

## Methods

### Plant materials

The tubers of *G. elata* with dormant flowers buds were cultivated in a greenhouse at the Institute of Medicinal Plant Development, Chinese Academy of Medical Sciences & Peking Union Medical College, Beijing. During May, at anthesis, flowers were pollinated by hand with pollen donated from separate individuals. At 20 days after pollination, capsules prior to dehiscence were collected for symbiotic culture.

### Mycorrhizal fungus preparation

The *Mycena* strain with good stimulating effect on symbiotic germination of *G. elata* deposited at the Institute of Medicinal Plant Development was used in this study (Guo and Xu 1990). The *Mycena* strain was first incubated on potato dextrose agar medium (PDA: potato 200 g L^−1^, glucose 20 g L^−1^, agar 12 g L^−1^, pH 5.2 before autoclaving) in darkness at 25 ± 1 °C for 7 days, then actively growing mycelium from the colony margin was used as the fungal inoculum. For the symbiotic germination of *G. elata*, the mycelia culture medium was prepared by mixing fallen leaves of *Quercus* with wheat bran at a ratio of 8:2 (v/v), then inoculated with the *Mycena* inoculum and placed in darkness at 25 ± 1 °C until leaves were fully colonized with fungal hyphae.

### Symbiotic seed germination

In a laminar flow hood, *Quercus* leaves fully colonized with fungal hyphae were first placed on water agar medium in a 9-cm diameter Petri dish. Then capsules were surface sterilized in a 1% NaClO solution for 20 min and cut open to remove seeds for inoculation. Approximately 100 seeds of *G. elata* removed from capsules were inoculated on the surface of each leaf, then dishes were sealed with paraffin and placed in darkness at 25 ± 1 °C. Seed germination and the formation of protocorms at each developmental stage were observed and recorded under a stereomicroscope weekly for 12 weeks. Germination was defined as emergence of the embryo from the seed coat (i.e., stage 2 as described by Stewart et al. 2003).

### Light microscopy

The seeds and developing mycorrhizal protocorms were fixed in a solution of 2.5% glutaraldehyde in 0.1 M phosphate buffer (pH 6.8) overnight at room temperature, washed in phosphate buffer three times and dehydrated with an ethanol series, then embedded in Technovit 7100 resin (Kulzer and Co., Germany) as described (Yeung and Chan 2015). Sections of 3 μm were cut by use of glass knives of the Reichert-Jung 2040 Autocut rotary microtome. For histological observations, sections were stained with Periodic acid–Schiff (PAS) reaction for total insoluble carbohydrates and counterstained with 0.05% (w/v) toluidine blue O (TBO) (Yeung 1984). Sections were observed and images were captured by using a CCD camera attached to a light microscope (Axio ImagerA1, Carl Zeiss AG).

### Transmission electron microscopy (TEM)

Symbiotic protocorms were collected and fixed by high-pressure freezing in a high-pressure freezer (Leica EM PACT2) as described (Li et al. 2018). The fixed protocorms were exposed to freeze substitution medium (ethanol containing 1% osmium tetroxide, 0.2% glutaraldehyde and 0.1% uranyl acetate) in a Leica Automatic Freeze-Substitution System, then embedded in London Resin White methacrylate resin (London Co., Basingstoke, UK). Ultrathin sections (70–90 nm) were cut by use of the diamond knife of the Leica Reichert Ultracut S system (Leica Microsystems GmbH1) and placed on formvar-coated nickel grids for observation under a Philip CM 100 transmission electron microscope (FEI Company2) at 80 kV.

## Results

**Fig. 1:**
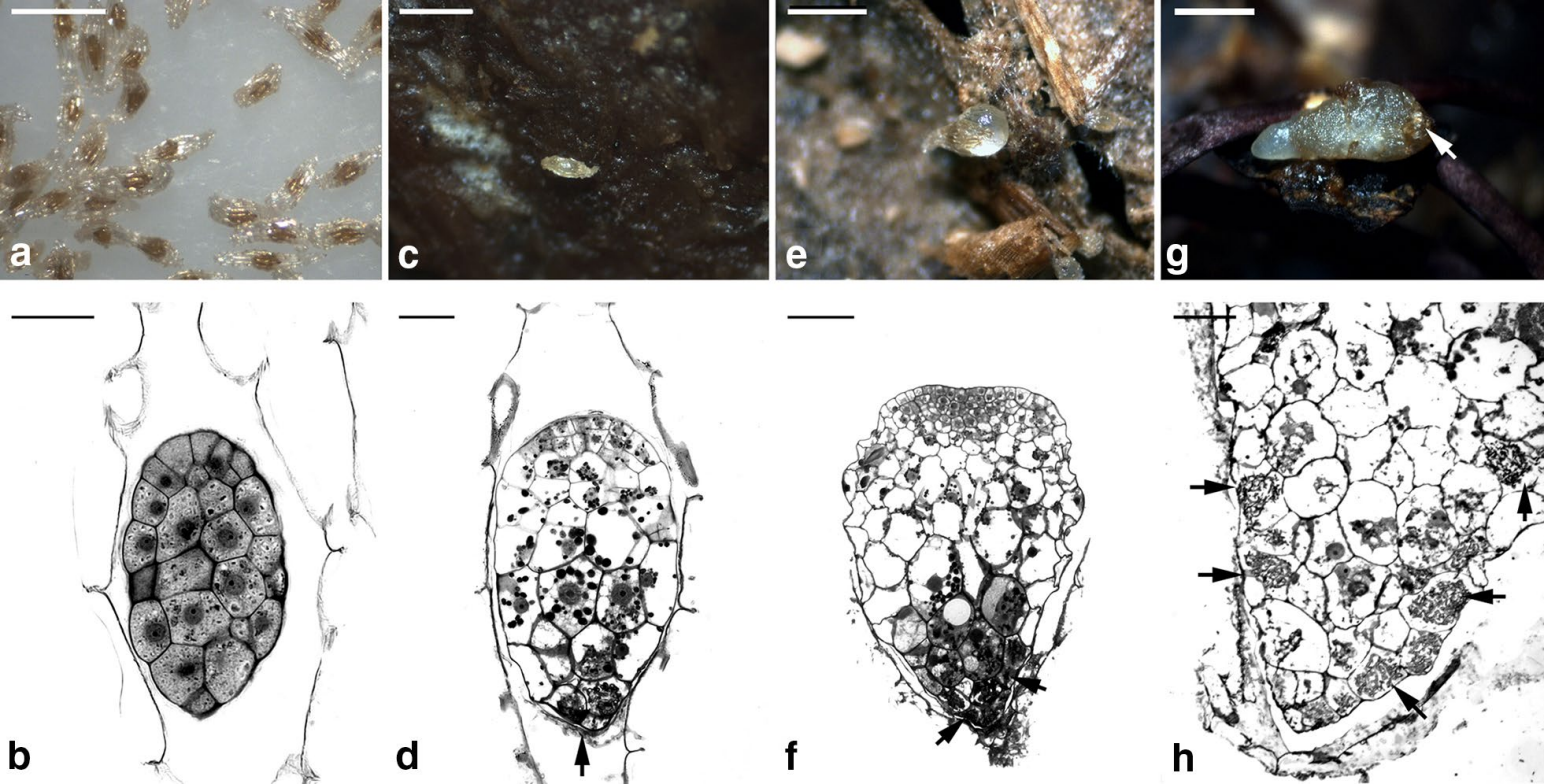
The symbiotic seed germination of *G. elata* associated with *Mycena*. **a** Mature seeds of *G*. *elata*. Scale bar = 0.5 mm. **b** Light micrograph of mature seed. The embryo is covered by a thin testa. Scale bar = 50 μm. **c** After 1 week of inoculation, a seed becomes swollen. Scale bar = 0.5 mm. **d** In the enlarged embryo, fungal hyphae have penetrated the embryo through the suspensor end cell (arrow). Scale bar = 50 μm. **e** After 2 weeks of inoculation, the embryo has ruptured the seed coat, resulting in the formation of a protocorm. Scale bar = 0.5 mm. **f** Fungal hyphae (arrows) have colonized the basal region of the developing protocorm. Scale bar = 100 μm. **g** After 12 weeks of inoculation, the elongated protocorm is observed. The basal region of the protocorm is indicated by an arrow. Scale bar = 1 mm. **h** Light micrograph showing the basal region of the elongated protocorm, and fungal hyphae (arrows) are restricted at the basal region. Scale bar = 60 μm

Mature seeds of *G. elata* had an oval-shaped embryo surrounded by a thin seed-coat layer (Fig. 1a, b). During the first week of inoculation, the embryo slightly enlarged and the embryo cells became highly vacuolated (Fig. 1c, d). By week 2 after inoculation, the embryo continued to enlarge, then ruptured the seed coat to form a protocorm (Fig. 1e, f). At this stage, the cells divided frequently at the apical part of protocorm to generate a meristematic zone, but cells at the basal part of protocorm did not further divide and were colonized by fungal hyphae. After 12 weeks of inoculation, the protocorm elongated further (Fig. 1g), and the fungal colonization was restricted to the basal protocorm (Fig. 1h).

**Fig. 2:**
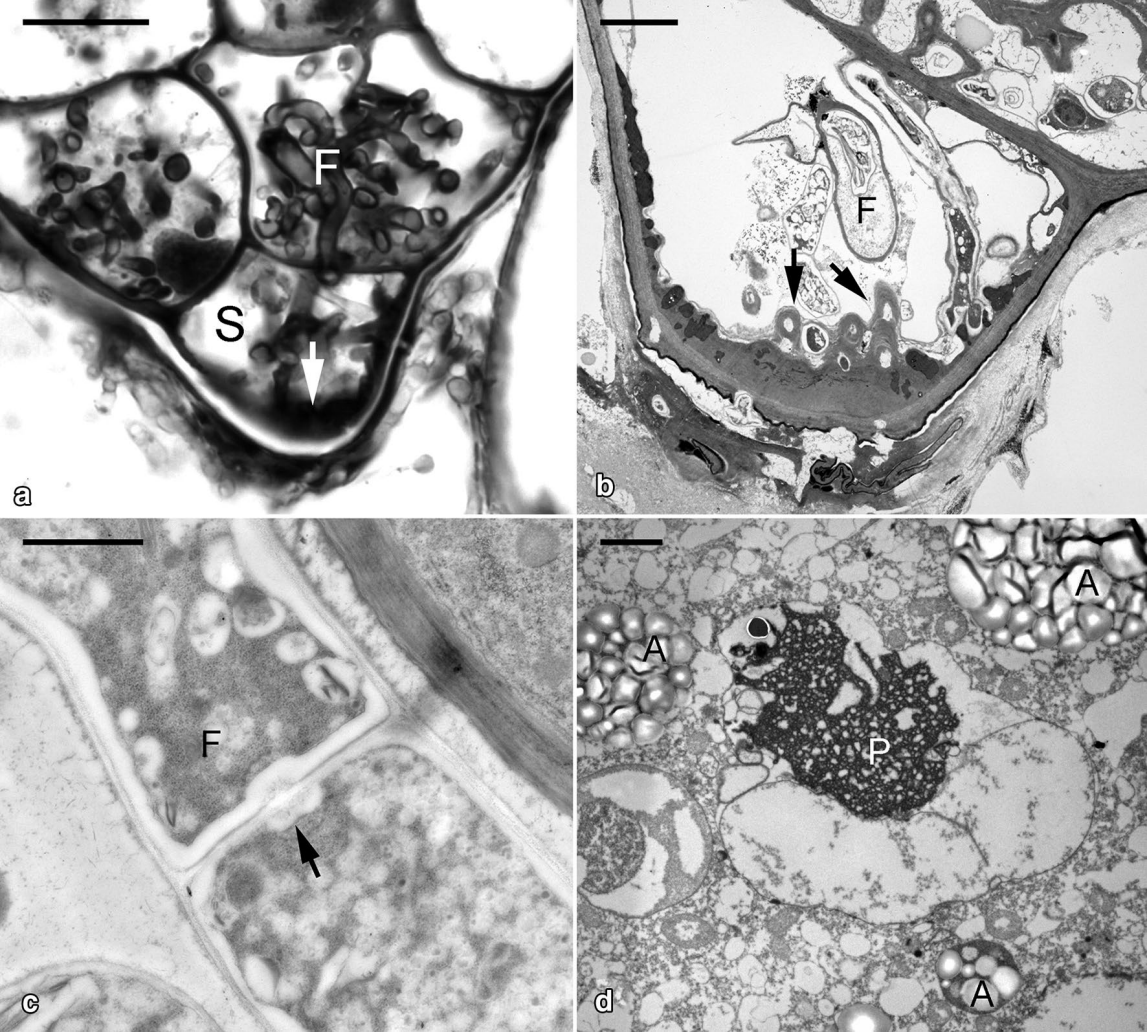
Micrographs showing the germinating embryo of *G. elata* associated with *Mycena*. **a** Light micrograph of the suspensor end cell (S) colonized by fungal hyphae (F) with cell wall thickening (arrow).Scale bar = 10 μm. **b** Ultrastructural view of the suspensor end cell showing the papillae-like cell wall thickening (arrows) corresponding to the entry of fungal hyphae. Scale bar = 2 μm. **c** At this stage, the intact fungal hypha (F) is present in the primarily colonized cells. The dolipore septum (arrow) can be observed at the junction between fungal cells. Scale bar = 1 μm. **d** In the uncolonized embryo cells, the storage protein bodies (P) are degrading and amyloplasts (A) start to appear. Scale bar = 4 μm

In the early stage of inoculation, fungal hyphae primarily penetrated the suspensor end of an embryo (Fig. 2a). The cell wall of the suspensor end became thickened substantially with the formation of papillae-like structures (Fig. 2b). Within the suspensor end cell, many fungal hyphae remained vigorous, but some were digested (Fig. 2b, c). In the uncolonized embryo cell, the protein bodies were degrading and amyloplasts began to appear (Fig. 2d).

**Fig. 3:**
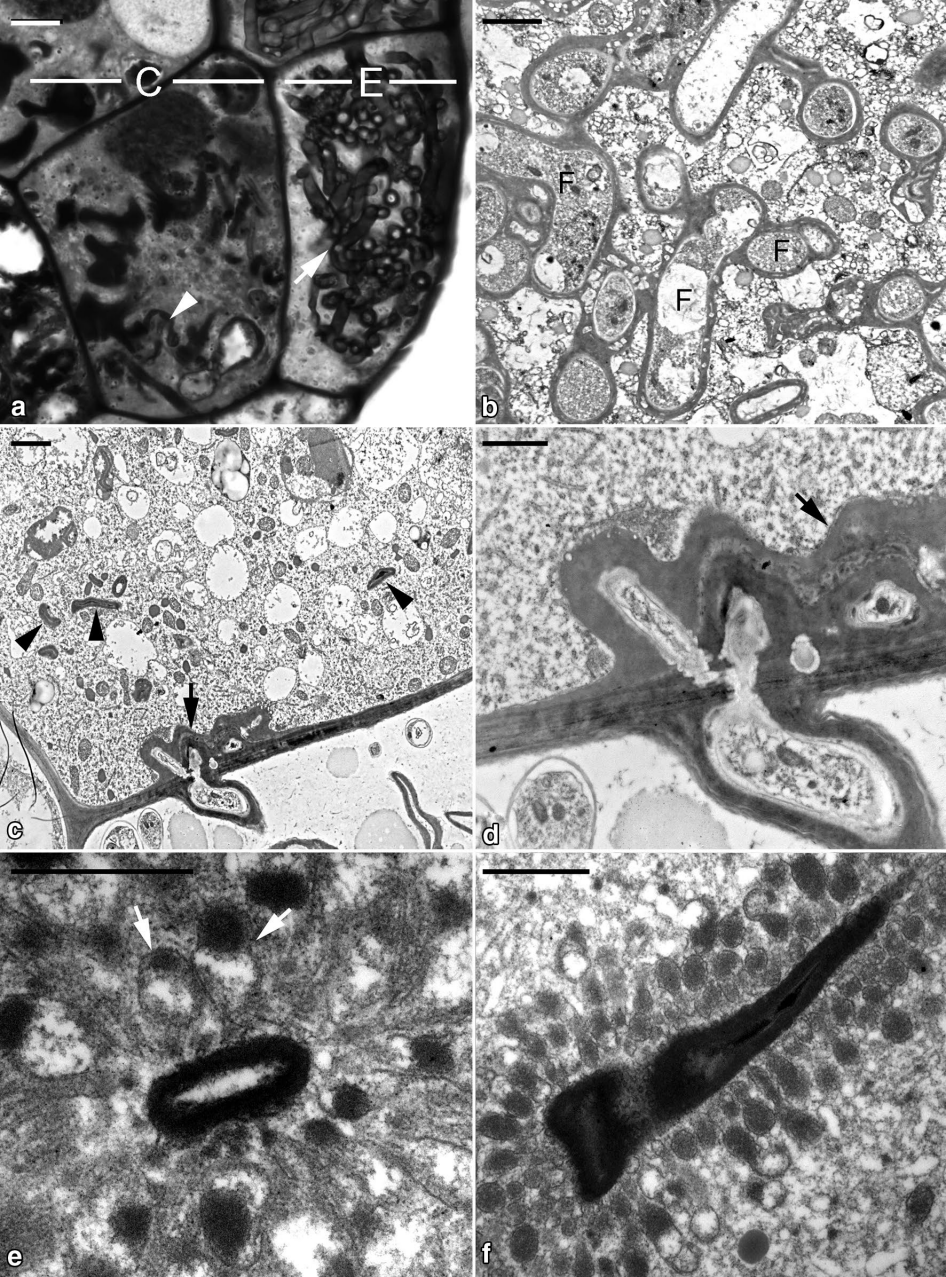
Micrographs showing the developing protocorm of *G. elata* associated with *Mycena*. **a** Light micrograph showing the colonized region of a developing protocorm. The epidermal cell (E) contains intact fungal hyphae (arrow), and digested fungal hyphae (arrowhead) are present in the cortical cell (C). Scale bar = 10 μm. **b** In the epidermal cell, a number of intact fungal hyphae (F) are present and are separated from the host cytoplasm by an enveloping interfacial matrix and host plasma membrane. Scale bar = 2 μm. **c** The penetration of fungal hyphae into the cortical cell (arrow). Inside the cortical cell, fungal hyphae are digested and become compressed (arrowheads). Scale bar = 2 μm. **d** After fungal hyphae penetrate the cortical cell, the fungal wall becomes thickened (arrow) by wrapping around additional material of the interfacial matrix and/or host plasma membrane cover. Scale bar = 1 μm. **e** In the cortical cell, several electron-dense endocytic tubules (arrows) attach a digesting fungal hypha. In cross sections, the tubular networks appear as numerous vesicles. Scale bar = 1 μm. **f** A compressed fungal hypha is surrounded by numerous electron-dense endocytic tubules. Scale bar = 1 μm

After seed germination, the embryo further enlarged, which resulted in the formation of an ovoid protocorm (Fig. 1e, g). In the basal region of the protocorm, the fungal hyphae coiled to form a loose peloton in epidermal cells, which was absent in cortical cells (Fig. 3a). In epidermal cells, most fungal hyphae remained active, and a few hyphae were digested (Fig. 3b). Penetration of fungal hyphae between the wall of epidermal cells and cortical cells was frequent (Fig. 3c). The fungal wall became thickened during penetration (Fig. 3d). After entry into the cortical cells, fungal hyphae were soon digested and became compressed. Ultrastructural observations indicated that numerous electron-dense tubular networks appeared and were associated with the surface wall of fungal hyphae (Fig. 3e, f). In cross sections, the tubular networks appeared as vesicles surrounding fungal hyphae (Fig. 3e).

**Fig. 4:**
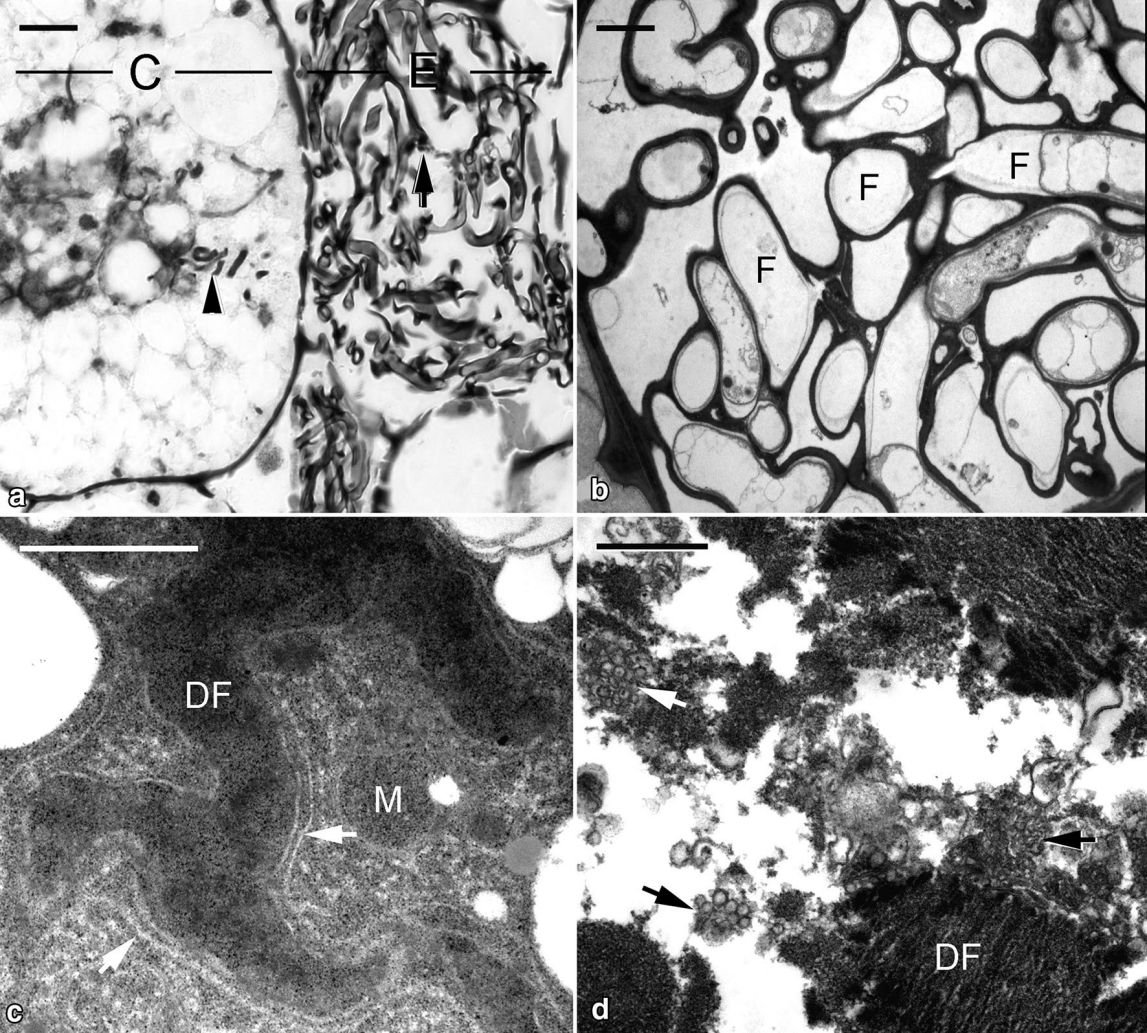
Micrographs showing the elongated protocorm of *G. elata* associated with *Mycena*. **a** Light micrograph showing the colonized region of the elongated protocorm. The epidermal cell (E) contains old fungal hyphae (arrow), and fragments of digested fungal hyphae (arrowhead) are visible in the cortical cell (C). Scale bar = 10 μm. **b** In the epidermal cell of the elongated protocorm, the cytoplasm of old fungal hyphae (F) has degenerated. Scale bar = 2 μm. **c** In the cortical cell of the elongated protocorm, the digested fungal hyphae (DF) are surrounded by rough endoplasmic reticulum (arrows) and a few mitochondria (M). Scale bar = 1 μm. **d** The digested fungal hyphae (DF) become fragmented and associated with clusters of vesicles (arrows). Scale bar = 1 μm

As the protocorm elongated further (Fig. 1h), the fungal hyphae became weak in the epidermal cells at the basal region of a protocorm (Fig. 4a). The cytoplasmic components of these fungal hyphae had broken down and the fungal wall became thickened in the epidermal cells (Fig. 4b). In cortical cells, the volume of vacuoles became larger, and a few compressed fragments of fungal hyphae were visible (Fig. 4a). At this stage, the compressed fungal hyphae assumed a dense appearance and were surrounded by rough endoplasmic reticulum (Fig. 4c). Subsequently, a number of clusters of vesicles appeared and were associated with the fragmented fungal hyphae (Fig. 4d).

## Discussion

In orchid mycorrhiza, fungal hyphae branch and coil, forming the structure called a peloton within parenchyma cells. Nutrients such as carbon and nitrogen are transferred between orchids and their fungal partners across the interfacial matrix, an apoplastic space produced between the fungal peloton and the orchid cell membrane (Kuga et al. 2014; Fochi et al. 2017). As the peloton is digested, orchids receive fungal nutrients from rupturing fungal hyphae. In our previous studies and the present investigation, the intracellular hyphae of *Mycena* in cortical cells are digested without forming peloton clumps (Fan et al. 1999; Fig. 3c). These observations suggest a mycorrhizal association between *G. elata* and *Mycena* belonging to the phytophagous type of orchid mycorrhiza.

The phytophagous type has been documented in only a few fully mycoheterotrophic orchids, in which fungal hyphae are lysed without forming peloton clumps, then release their cellular contents for use by the orchid (Burgeff 1936; Rasmussen 2002). However, in previous observations, the endocytic vesicles are absent around the lysed fungal hyphae in the symbiotic germination with *Mycena* (Fan et al. 1999, 2001). One of the notable findings in this study is the presence of tubular endocytic networks attached to the lysed fungal hyphae in the cortical cells of symbiotic protocorms (Fig. 3e, f). During the symbiotic germination (e.g., stages 2 and 3), the fungal hyphae of *Mycena* from dermal cells penetrate cortical cells and are surrounded by tubular endocytic networks. This structural feature is identical to the phytophagous type of orchid mycorrhiza association characterized in the association of *G. elata* with *Armillaria* (Wang et al. 1997; Rasmussen 2002). After the symbiotic germination with *Mycena*, the growth tube of *G. elata* requires a switch to a mycorrhizal association with *Armillaria*. In the inner cortical cells of the growth tube, *Armillaria* hyphae appear to be lysed as the phytophagous type of orchid mycorrhiza. Ultrastructural studies indicate that as hyphae enter an enlarged digestion cell, the plant plasmalemma and fungal wall (the interfacial matrix) are surrounded by the radiating endocytic tubules to mark the final stage of fungal hyphae breakdown (Wang et al. 1997). Staining with acid phosphatase also suggested the digestive role of these electron-dense tubular networks in cortical cells of *G. elata* tubers (Wang and Xu 1993).

The lack of endocytic tubules in our previous studies of the symbiotic germination with *Mycena* is probably due to the degraded tubular networks during sample fixation. In the present study, the mycorrhizal protocorms were prepared by a high-pressure freezing technique, and the dynamic organelle structures were well preserved. A recent transcriptome analysis of *G. elata* in symbiotic germination with *Mycena* revealed high up-regulation of a number of genes involved in endocytosis (Zeng et al. 2017). The observations in this study provide the supportive ultrastructural data and suggest nutrient transfer via endocytosis in the phytophagous type of orchid mycorrhiza.

Although the mycorrhizal protocorm of *G. elata* is small and simple, histology revealed two distinct cell types in the colonization region: a epidermal cell with a degraded cytoplasm and nucleus and filled with actively growing fungal hyphae and a cortical cell with a dense cytoplasm containing lysed fungal hyphae (Fig. 3a). The epidermal cell of the mycorrhizal protocorm is similar to the passage canal cell in the outer cortex of the mycorrhizal tuber in the association with *Armillaria*, and the cortical cell of the mycorrhizal protocorm resembles the enlarged digestion cell in the inner cortex of the mycorrhizal tuber (Wang et al. 1997). In *Gastrodia*, the differentiation of the two cell types in mycorrhizal tissue may be an adaptation to these litter- and wood-decaying fungal partners.

Papillae-like cell wall thickening was apparent in walls of epidermal cells of the mycorrhizal protocorm (Fig. 2b) and passage canal cells of the mycorrhizal tuber (Jonsson and Nylund 1979). These cell wall thickenings also mark the penetration sites of fungal hyphae, especially in the suspensor end cell and the adjoining walls between the epidermal and cortical cells (Figs. 2b, 3c). Similar papillae-like cell wall thickening was found in mycorrhizal roots of other *Gastrodia* species (Martos et al. 2009; Lee et al. 2015). The cell wall thickening could be a defense response to the fungal invasion (Voigt 2014). Moreover, these cells with papillae-like cell wall thickening may be specialized transfer cells in the network of nutrient transport in the mycorrhiza (Pate and Gunning 1972). Further studies of the dynamic changes of cell wall proteins would provide insights into the possible role of the cell wall thickening in mycorrhizal tissue of *Gastrodia* species.

## Conclusions

The present study provides critical evidence for the phytophagous type of orchid mycorrhiza in symbiotic seed germination of *G. elata* with *Mycena*. In symbiotic protocorms, the presence of the passage canal cell in the epidermal cell, the digestion cell in the cortex, and the endocytic tubules for hyphae digestion demonstrate a particular nutrient transfer network in the association with litter-decaying fungal partners.

## Data Availability

Not applicable.
